# Meditation and yoga practice are associated with smaller right amygdala volume: the Rotterdam study

**DOI:** 10.1007/s11682-018-9826-z

**Published:** 2018-02-07

**Authors:** Rinske A. Gotink, Meike W. Vernooij, M. Arfan Ikram, Wiro J. Niessen, Gabriel P. Krestin, Albert Hofman, Henning Tiemeier, M. G. Myriam Hunink

**Affiliations:** 1 Department of Epidemiology, Erasmus Medical Center, Rotterdam, the Netherlands; 2 Department of Radiology, Erasmus Medical Center, Rotterdam, the Netherlands; 3 Department of Medical Informatics, Erasmus Medical Center, Rotterdam, the Netherlands; 4 Department of Epidemiology, Harvard T.H. Chan School of Public Health, Boston, MA USA; 5 Department of Psychiatry, Erasmus Medical Center, Rotterdam, the Netherlands; 6 Department of Social and Behavioral Sciences, Harvard T.H. Chan School of Public Health, Boston, MA USA; 7 Center for Health Decision Sciences, Harvard T.H. Chan School of Public Health, Boston, MA USA

**Keywords:** Cohort, Meditation and yoga, Neuroimaging, Population, Stress reduction

## Abstract

To determine the association between meditation and yoga practice, experienced stress, and amygdala and hippocampal volume in a large population-based study. This study was embedded within the population-based Rotterdam Study and included 3742 participants for cross-sectional association. Participants filled out a questionnaire assessing meditation practice, yoga practice, and experienced stress, and underwent a magnetic resonance scan of the brain. 2397 participants underwent multiple brain scans, and were assessed for structural change over time. Amygdala and hippocampal volumes were regions of interest, as these are structures that may be affected by meditation. Multivariable linear regression analysis and mixed linear models were performed adjusted for age, sex, educational level, intracranial volume, cardiovascular risk, anxiety, depression and stress. 15.7% of individuals participated in at least one form of practice. Those who performed meditation and yoga practices reported significantly more stress (mean difference 0.2 on a 1–5 scale, p < .001) and more depressive symptoms (mean difference 1.03 on CESD, p = .015). Partaking in meditation and yoga practices was associated with a significantly lower right amygdala volume (β = − 31.8 mm^3^, p = .005), and lower left hippocampus volume (β = − 75.3 mm^3^, p = .025). Repeated measurements using linear mixed models showed a significant effect over time on the right amygdala of practicing meditation and yoga (β = − 24.4 mm^3^, SE 11.3, p = .031). Partaking in meditation and yoga practice is associated with more experienced stress while it also helps cope with stress, and is associated with smaller right amygdala volume.

## Introduction

Mind–Body practices are becoming increasingly popular in both supporting medical treatment of chronic patients and prevention of disease in the healthy population (Wolsko et al. 2004; Barnes et al. 2008). Approximately 20% of the US population use mind–body practices, such as meditation and yoga, to gain a more active role in their health. Mindfulness for instance teaches awareness of bodily sensations of stress and of reaction patterns in a non-judgmental manner. Meditation and yoga have been shown to reduce anxiety, depression and stress, and to improve quality of life (Gotink et al. 2015; Lin et al. 2011). Unfortunately, there is no clear understanding of how these practices exert their positive effect. A recent meta-analysis showed that cognitive and emotional reactivity are the largest mediators underlying the improvements, together with mindfulness, rumination, self-compassion and psychological flexibility (Gu et al. 2015). Previous neuroimaging meta-analyses on traditional meditation styles (i.e. Zen, Vipassana, Tibetan etc.) show that long term meditators consistently exhibit a different gray matter morphometry in several regions: the prefrontal cortex (PFC) (which is related to attentional processes), the sensory cortices and insula (related to body awareness), the hippocampus (related to memory processes), the cingulate cortex (related to self and emotion regulation), and the amygdala (related to the fight-flight response) (Lazar et al. 2005; Pagnoni and Cekic 2007; Holzel et al. 2008; Luders et al. 2009; Vestergaard-Poulsen et al. 2009; Tomasino et al. 2013; Fox et al. 2014). A review on neuroimaging in yoga practitioners also showed decreased blood flow in the amygdala and increased activity in the prefrontal cortex, suggesting that practitioners do notice negative stimuli, but are less affected by it (Desai et al. 2015).

The amygdala seems to be typically involved in stress and anxiety: related to instinct and the fight-flight reaction, it adds emotional value to sensory input (Kolb and Wishaw 2009) and functions as a pre-consciousness warning system (Craigmyle 2013). Stress reduction has been associated with less amygdala volume (Holzel et al. 2010), and research has found significant differences in amygdala activity and structure in connection with both training and pre-dispositional mindfulness (Creswell et al. 2007; Modinos et al. 2010; Taren et al. 2013; Lutz et al. 2013). Meditation shows also an improved memory function and meditators have larger hippocampal volumes than controls (Murakami et al. 2012; Pickut et al. 2013; Holzel et al. 2011). So it seems that these neural structures could be affected by the stress reducing effects of meditation and yoga. The amygdala and the hippocampus are part of an extended neural network (Phelps et al. 2004); earlier studies on the effect of meditation also showed a stronger connectivity between the ventromedial PFC and the amygdala, where the former down-regulated the activity of the latter (Modinos et al. 2010; Holzel et al. 2013) and an increase in activity in the hippocampus (Goldin and Gross 2010; Creswell et al. 2007). However, all these previous studies have been performed either in specific patient populations or in small samples of healthy, young volunteers. No study has yet been conducted in a large population-based setting. Doing so would give greater insight in practitioner characteristics in real-life, and whether these methods are effective in an uncontrolled population setting rather than a controlled clinical setting. Also, it could give insight in practice associated mental health, and in underlying neuronal differences between practitioners and non-practitioners.

The current study investigates whether the relationship between meditation and yoga practice and experienced stress levels, is associated with differences in amygdala and hippocampal volumes in a large Dutch population-based sample of middle aged and elderly subjects.

## Methods

### Study population

The Rotterdam Study is an ongoing population-based cohort in the Netherlands that aims to investigate causes and determinants of diseases in elderly adults (Hofman et al. 2015). Recruitment began in 1990, and the current study population consisted of 14,926 subjects 45 years and older at baseline. The whole cohort undergoes physical and psychological re-examinations every two to three years. Since 2005, all participants of the Rotterdam Study without contraindications to Magnetic Resonance Imaging (MRI) are invited to undergo a brain MRI examination as part of the Rotterdam Scan Study, which aims to investigate causes and consequences of age-related brain changes (Ikram et al. 2011).

**Fig. 1:**
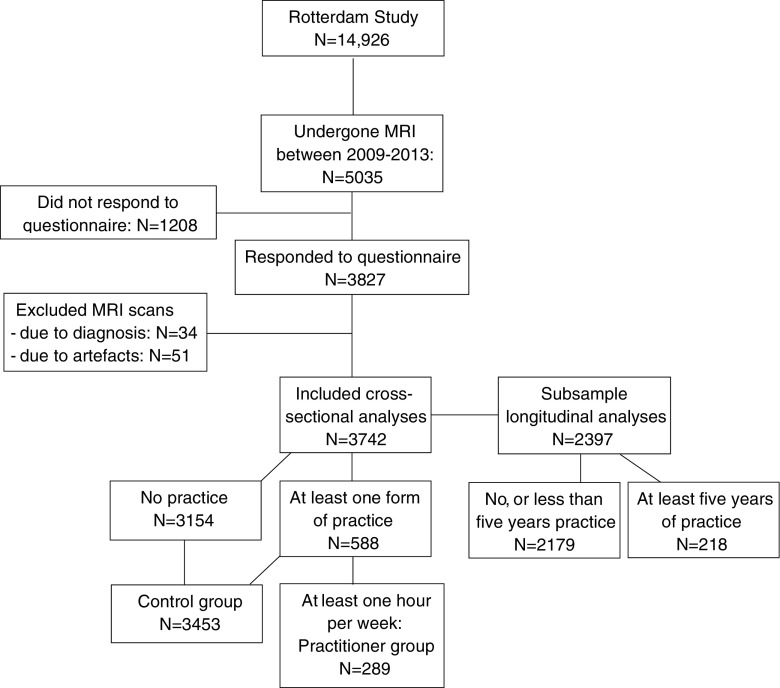
Flow chart of participant selection

For our study, we included individuals examined at the research center between 2008 and 2013. We included all participants that responded to a meditation and yoga practice questionnaire and had undergone an MRI of the brain (n = 3827). Diagnosis of clinical stroke, dementia and Parkinson were reason for exclusion (n = 34). After removing uninterpretable MRI scans, 3742 participants were included for the cross-sectional analyses. We also analyzed a subgroup that underwent a brain scan 5 years earlier as part of the Rotterdam Study, to see whether there was a difference in brain structure changes between the groups over time. For this we allocated participants to the practitioner group if they practiced for 5 years or longer (see Fig. 1).

The Rotterdam Study was approved by the medical ethics committee according to the Population Study Act Rotterdam Study, executed by the Ministry of Health, Welfare and Sports of the Netherlands. Written informed consent was obtained from all participants.

### Measurement of meditation and yoga practices

During a one-time home interview, trained interviewers questioned individuals if they performed meditation and yoga practices, which were defined as ‘looking for stillness inside oneself’, ‘meditation’ or ‘mindfulness’ (captured in this article under ‘meditation’), ‘yoga’, ‘Tai Chi’, ‘Qi Gong’ or ‘similar movement therapy’ (captured under ‘yoga’), and relaxation or breathing exercises (referred to as ‘breathing exercises’). As breathing exercises are such a core aspect of both meditation and yoga, this category is included without being named separately in this article. Additionally, the interview contained a question whether practicing this particular technique helped participants in coping with stress. The questionnaire also included the amount of time per week spent on the practice (0 h, < 1 h, 1–2 h, > 2 h), and the number of years of practice. For the exposed group, a cut-off of at least one hour per week was chosen in order to assess the effect of practice, and at least one year of practice. Participants practicing less than 1 h per week or less than a year (N = 239) were allocated to the control group.

### Brain MRI acquisition and post-processing for amygdala volume

Brain MRI data were acquired with a 1.5-Tesla scanner (GE Healthcare, Milwaukee, Wisconsin) using an eight-channel head coil during a 30-min brain imaging protocol that was previously described in detail (Ikram et al. 2011; Jones et al. 1999). Two trained technicians performed all examinations in a standardized way. This protocol included high-resolution axial fluid-attenuated inversion recovery (FLAIR), T1-weighted, and T2-weighted sequences. The T1-weighted image was used for amygdala segmentation and consisted of a 3D spoiled gradient-recalled echo (SPGR) scan, with voxel volume of 0.49 × 0.49 × 0.80 mm^3^. Automatic segmentation of subcortical brain structures, including the amygdala and hippocampus left and right, was performed on T1-weighted images using Freesurfer software (version 4.5.0) (FreeSurfer 2013). This rendered volumetric measures of gray matter (in mm^3^). Exact processing details are described elsewhere (Reuter et al. 2012; Desikan et al. 2006; Fischl et al. 2002).

### Co-variables

In order to assess the relationship between meditation and yoga practice, and amygdala and hippocampal volume, we took demographic variables, total intracranial volume (ICV in mm^3^, derived from automated brain segmentation), cardiovascular risk factors and psychological functioning into account as variables that can otherwise affect brain structure measurements. Highest education achieved was categorized as “primary education”, “lower and intermediate general education or lower vocational education”, “intermediate vocational education or higher general education”, or “higher vocational education or university”, according to the UNESCO guidelines (Statistics 2012).

Cardiovascular risk factors that were taken into account were body-mass index (BMI), systolic and diastolic blood pressure, antihypertensive medication, total serum cholesterol, high density lipoprotein cholesterol, triglycerides, low-density lipoprotein-cholesterol calculated with the Friedewald Formula (Friedewald et al. 1972), diabetes mellitus, smoking and use of alcohol in the assessment of the amygdala volume, see for exact measurement methods elsewhere(Mosterd et al. 2001). Smoking status was categorized as never, former, and current smoker. Alcohol use was categorized as never, regular use (> 2–3 times per week at least 1 beverage), and occasional use (< 2–4 times per month at least 1 beverage).

Psychological functioning was assessed during home interviews with validated questionnaires and comprises measurements of stress, depression and anxiety. The level of experienced stress was assessed with a 1 item 5-point Likert scale (“how much stress do you experience on a scale from 1 (not at all) to 5 (very much)?”). Stress unfortunately was measured only once, so no longitudinal data was available. To identify current depressive symptoms, we used the Center for Epidemiological Studies Depression (CES-D) scale, a 20-item questionnaire which has been validated in a variety of populations (Lewinsohn et al. 1997). Anxiety was measured with the seven anxiety items of the Hospital Anxiety and Depression Scale (HADS-A) (Spinhoven et al. 1997). These items are scored on a 4-point scale ranging from 0 (absence of the symptom) to 3 (extreme presence), the sum score indicates the anxiety level.

### Statistical analysis

For the cross-sectional analyses, ANCOVA analyses were performed to compare characteristics of individuals in the exposed group to the control group, and for the three practitioners’ subgroups for exploratory indications. Apart from demographics, all other variables were adjusted for age, sex and education level. All imaging outcomes were also adjusted for ICV. Subsequently, we conducted multivariable linear regression analyses to evaluate the association between meditation and yoga practice, and left and right amygdala volume. As literature indicates that cortical thickness increases with meditation practice (Lazar et al. 2005; Grant et al. 2010; Kang et al. 2013), we also performed an analysis on the ratio between amygdala volume and total brain volume. For the longitudinal analyses, linear mixed models were used adjusted for all covariables to account for repeated measurements.

### Imputation

2 percent of values were missing, none of which concerned outcome variables. Multiple imputation was performed using 10 iterations with all variables as predictors. In the variables assessing how many years participants practiced their activity, missing values due to remembrance issues (“I don’t remember how many years I have been practicing”) were imputed by the median value of the group to account for right censoring. A p-value less than 0.05 was considered to indicate statistical significance. All data were analyzed with IBM SPSS Statistics version 21.0 (IBM Corp 2012).

## Results

### Participant characteristics

Mean age at the time of the MRI scan was 64.1 years (SD 7.7) and 55% were women. A total of 588 individuals (15.7%) reported engaging in any form of practice. Using a cut-off of 1 h per week, 289 individuals performed a practice (Table 1) with meditation being the most often performed practice (N = 159). 98 participants practiced yoga or similar movement therapy, and breathing exercises were done by 75 participants (Table 2). 43 individuals took part in more than one practice. Compared to those who did not report partaking in meditation and yoga, individuals who did a form of practice were higher educated, significantly more often female (73%) and younger (61.9 years, SD 6.8) (Table 1). The median duration of practice in the exposed group was 10 years.

**Table 1:** Characteristics of the study population

	No meditation and yoga practices (N = 3453)	Meditation and yoga practices (N = 289)
Mean age, years (SD)	64.3 (7.7)	61.9 (6.8)^a^
Women	53%	73%^a^
Highest education, score (SD)	1.7 (0.9)	2.0 (0.9)^a^
BMI (SD)	27.5 (4.3)	26.6 (4.4)^a^
SBP mmHg (SD)	139.7 (19.9)	138.3 (20.3)
DBP mmHg (SD)	83.2 (11.3)	82.9 (11.6)
Diabetes mellitus	8.3%	6.4%
HDL cholesterol, mmol/L (SD)	1.5 (0.4)	1.5 (0.4)
LDL cholesterol, mmol/L (SD)	3.7 (1.1)	3.8 (1.0)
Smoking score (SD)	0.43 (0.6)	0.45 (0.7)
Alcohol score (SD)	1.45 (0.6)	1.48 (0.8)
Stress (1–5), mean (SD)	2.0 (1.0)	2.2 (1.0)^a^
Depression, mean (SD)	5.2 (6.8)	6.2 (6.9)^a^
Anxiety, mean (SD)	15.8 (3.4)	15.4 (3.5)
Total brain, ml (SD)	940.4 (44.3)	943.1 (45.0)
Left amygdala, mm^3^ (SD)	1334.8 (170.9)	1321.4 (173.5)
Right amygdala, mm^3^ (SD)	1415.8 (169.4)	1386.8 (172.0)^a^
Left hippocampus, mm^3^ (SD)	3958.1 (511.3)	3891.2 (519.0)^a^
Right hippocampus, mm^3^ (SD)	3959.6 (496.1)	3920.9 (503.5)

Analyses of lifestyle, cardiovascular risk factors and psychological measures were performed adjusted for age, sex and education level. Analyses of brain volumes were also adjusted for ICV

^a^Significantly different (p < .05). Groups are based on a cut-off of at least 1 h per week practice

1 ml = 1000 mm^3^

**Table 2:** Types of practice, mean duration and hours spent per week

	Meditation	Yoga	Breathing exercises
N 0–1 h per week	96	37	106
N 1–2 h per week	83	69	36
N > 2 h per week	76	29	39
Mean duration, years (SD)	14.9 (15.5)	11.2 (10.6)	13.7 (13.2)
N (%)^a^	159 (55%)	98 (34%)	75 (26%)

Some participants performed more than one practice (e.g. both meditation and yoga. Unique N = 289, unique total N = 588)

^a^Cut-off: practice 1 h or more per week

### Meditation and yoga practices and mental health

Stress and depression were rated significantly higher among practitioners, while 90.7% of practitioners reported that doing their activities helped them cope with stress (5.5% reported it did not and 3.8% did not know). Anxiety did not differ significantly between practitioners and non-practitioners. Analyzing subgroups, current depressive symptoms were rated significantly higher amongst meditators but not in yoga or breathing exercise practitioners, whereas stress was significantly higher in the yoga and breathing exercise groups but not in meditators.

### Cross-sectional analyses: meditation and yoga practices and amygdala and hippocampal volume

Total brain volume was not different between those practicing compared to controls (943.1 ml versus 940.4 ml, p-value = 0.32) (see Table 1). The left amygdala was not different between the two groups (1321.4 mm^3^ in the practitioners versus 1334.8 mm^3^ in the controls (age-, sex-, education- and ICV-adjusted, p-value = 0.21)); but the right amygdala was: 1386.8 mm^3^ versus 1415.8 mm^3^ (p < .01). The ratio between right amygdala and total brain volume was significantly smaller in practitioners than in non-practitioners (0.147% versus 0.151%, p-value = 0.001). Comparing subgroups, though all of these differences were not statistically significant, the right amygdala was smallest in the meditation group, whereas the left was smallest amongst the breathing exercise participants, the yoga group showed the largest amygdalae. The ratio between right amygdala and total volume was significantly smaller in the meditation and breathing groups compared to the control group, though be it minimal (differences 0.003% p = .018 and 0.004% p = .046 respectively). The right hippocampus was not significantly different between groups. The left hippocampus, however, was significantly smaller in the practitioner group (3891.2 mm3) compared to the control group (3958.1 mm3), p = .036.

Linear regression analyses of the left and right amygdala volume with adjustment for demographics, intracranial volume, cardiovascular risk and psychological functioning resulted in the models shown in Table 3. Practicing meditation or yoga compared to not was associated with lower right amygdala volume (β = − 31.8 mm^3^, SE 11.2, p = .005). The left amygdala showed no significant association (β = − 14.5 mm^3^, SE 11.1, p = .192). Practice was associated significantly with the ratio between right amygdala and total brain volume (β = − 0.38%, SE 0.116, p = .001). Linear regression also showed that practice was associated with a smaller left hippocampus: β = − 75.3 mm^3^, SE 33.6, p = .025. Differences in the right hippocampus were not statistically significant.

**Table 3:** Cross-sectional analyses. Association between meditation and yoga practice and different brain volumes (N = 3742)

	β (mm^3^)	SE	p-value	95% Confidence interval	
				Lower	Upper
Total brain (mm^3^)	1352.6	2922.0	0.643	− 4374.4	7079.6
Left amygdala (mm^3^)	− 14.5	11.1	0.192	− 36.3	7.3
Right amygdala (mm^3^)^a^	− 31.8	11.2	0.005	− 53.8	− 9.8
Left hippocampus (mm^3^)^a^	− 75.3	33.6	0.025	− 141.1	− 9.5
Right hippocampus (mm^3^)	− 36.8	32.8	0.262	− 101.0	27.4

Regression analyses adjusted for age, sex, ICV, education, cardiovascular risk factors, depression, anxiety, and stress

^a^Significantly different (p < .05)

Depression score was significantly correlated with right amygdala volume in the control group (r = − 0.09, p < .001), but not in the practitioner group (r = − 0.10, p = .078). Stress was not significantly correlated with right amygdala volume (r = − 0.02, p = .227), but it was with practice (r = 0.09, p < .001). However, the amount of practice was not correlated with amygdala volume, depression or stress. When changing the cut-off to 2 h practice a week, there was no significant difference between the groups anymore.

### Longitudinal analyses

For analyzing structural change over time, 2397 participants were included who had MRI scans taken 5 years apart. In this sample, only 9% (N = 218) practiced meditation or yoga for 5 years or longer. Linear mixed models adjusted for all covariates showed a significant interaction over time of practicing meditation and yoga on right amygdala volume (β = − 24.4 mm^3^, SE 11.3, p = .031) (Table 4). When excluding the adjustment for psychological functioning from the model, the right amygdala showed a similar association (− 24.7 mm^3^, p = .028). There was no effect of amount of practice (frequency per week x years of practice) on right amygdala volume (β = − 0.4 mm^3^, SE 0.3, p = .15). The left amygdala did not show a significant association (β = − 11.8 mm^3^, SE 11.7, p = .312), nor did right and left hippocampus (β = 116.5 mm^3^, SE 84.6, p = .17 and β = − 59.4 mm^3^, SE 87.1, p = .50 respectively).

**Table 4:** Longitudinal analyses of the effect of meditation and yoga practice on volume over 5 years follow-up (N = 2397)

Parameter	β (mm^3^)	SE	p-value	95% Confidence interval	
				Lower	Upper
Left amygdala	− 11.8	11.7	0.312	− 34.7	11.1
Right amygdala^a^	− 24.4	11.3	0.031	− 46.5	− 2.3
Left hippocampus	− 59.4	87.1	0.495	− 230.6	111.7
Right hippocampus	116.5	84.6	0.169	− 49.7	282.7

Linear Mixed models adjusted for age, gender, education, ICV, cardiovascular risk factors, depression, anxiety, and stress

^a^Significantly different (p < .05)

Depression scores showed an increase over 5 years with similar effects in practitioners and controls (0.6 and 1.0 on the CESD respectively, p = .73). Furthermore, elucidating the causal relationship between higher depression scores and smaller amygdala volume, there was no effect of right amygdala volume on CESD score (β = − 0.001, SE 0.001, p = .21).

## Discussion

In this large population-based study we demonstrated that individuals involved in meditation and yoga practice experienced higher stress levels, whereas brain imaging shows smaller right amygdala and left hippocampal volume compared to those not practicing. These results are in line with previous correlational research amongst healthy participants (Taren et al. 2013). Practicing meditation and yoga had a significant relation over time with right amygdala volume, but not with left hippocampal volume, which is in line with longitudinal research (Holzel et al. 2011).

Elucidating the causal pathways of meditation and yoga practice, stress and amygdala volume in this context is complex. Early life stressors seem to have an increasing effect in later life on amygdala volume, and a decreasing effect on hippocampal volume (Tottenham and Sheridan 2009). Also, depression has been associated with a larger right amygdala volume (Lange and Irle 2004; Holzel et al. 2010). In the present study, we observed that the meditation and yoga group had a smaller right amygdala. Stress reduction has been associated with less amygdala volume (Holzel et al. 2010), and many other studies support this relationship (Farb et al. 2007; Goldin and Gross 2010; Desbordes et al. 2012; Creswell et al. 2007; Holzel et al. 2013). It therefore seems plausible that the found smaller amygdala volumes are due to less experienced stress through meditation practice. However, increasing the cut-off to 2 h of practice a week diminished the found association, presumably due to merging the smaller amygdala volume in the 1–2 h practitioner group with the non-practitioners. This would imply that practicing more hours a week does not per se increase the effect of practicing meditation and/or yoga. Also, there is a risk of confounding by indication, which can obscure the effect of meditation and yoga practice on psychological functioning (Grobbee and Hoes 2015). Meditation and yoga practices are known for their stress-reducing intent, making it plausible that people experiencing stress make use of these strategies, and that practice could even be seen as a marker of stress. However, not all persons experiencing stress choose meditation or yoga as coping strategy, and not all practitioners have started this practice because of stress. Furthermore, the regression analyses showed that with and without adjustment for stress, depression and anxiety, the association with a smaller right amygdala remains. This indicates that confounding by indication does not explain the differences in amygdala volume between the two groups. The higher reported stress and depression levels could also mean that meditation and yoga practitioners have become more aware of their stress, but are at the same time more able to deal with it hence the smaller amygdala volume.

Volumetric differences were only found in the right amygdala, not in the left. This is in line with previous smaller studies and is explained by the fact that the right amygdala, as opposed to the left amygdala, is associated with negative emotions and immediate action taking, whereas the left is associated with positive emotions and memory (Lanteaume et al. 2007; Murray 2009; Markowitsch 1998).

A number of limitations of this study deserve attention. Although performed in a very large population-based sample, this is still a selection of participants that are generally healthy and motivated to join research. The prevalence of meditation and yoga practice is slightly lower in this study population (15.7%) than in other studies (18.9%), where the latter population was notably younger (Wolsko et al. 2004). It could be that people with a smaller right amygdala are naturally drawn to meditation and yoga practices. There is also risk of information bias since the questionnaire assessed amount of practice retrospectively. Also, stress was measured with a one-item Likert-scale instead of a validated questionnaire, which can increase the risk of information bias due to inaccuracy. Furthermore, this study contains mainly elderly individuals, who may not be as actively involved in meditation and yoga practices as younger people might, and who also might show a different structural response than younger participants due to decreased brain plasticity. Physical activity is likely an intermediary in this association, and was therefore not taken into account, as causal inference methodology suggests (Pearl 2009). Finally, this study took a broad approach to the intervention of interest: practice involved meditation, yoga and breathing exercises, which in turn can comprise different styles.

Despite the limitations, the results of this large population-based study do give direction for future research on the stress reducing effects of lifestyle interventions. Seeing that natural behavior (as it concerns population-based observations) following these increasingly popular practices is aligned with the smaller scientific settings of earlier research, poses both encouragement to the application of the practices and a more detailed understanding of the neuronal working mechanism. Meditation and yoga practice are associated with structural differences in right amygdala volume and the vast majority of practitioners report that it helps them cope with stress. This suggests that meditation and yoga practices might be a feasible and accessible lifestyle intervention for people suffering from stress and stress-related diseases. Such practices could be helpful in prevention of stress-related diseases, by recognizing early stages of stress and changing the neural response to stressful stimuli.

## Conclusion

Partaking in meditation and yoga practice is associated with more experienced stress while it also helps cope with stress, and is associated with smaller right amygdala volume.

## References

[CR1] Barnes, P. M., Bloom, B., & Nahin, R. L. (2008). Complementary and alternative medicine use among adults and children: United States, 2007. *National Health Statistics Report* (12), 1–23. 19361005

[CR2] Corp, I. (2012). *IBM SPSS statistics for windows*. (21.0 ed.). Armonk, NY.

[CR3] Craigmyle NA (2013). The beneficial effects of meditation: contribution of the anterior cingulate and locus coeruleus. Frontiers in Psychology.

[CR4] Creswell JD, Way BM, Eisenberger NI, Lieberman MD (2007). Neural correlates of dispositional mindfulness during affect labeling. Psychosomatic Medicine.

[CR5] Desai R, Tailor A, Bhatt T (2015). Effects of yoga on brain waves and structural activation: a review. Complementary Therapies in Clinical Practice.

[CR6] Desbordes G, Negi LT, Pace TW, Wallace B, Raison CL, Schwartz EL (2012). Effects of mindful-attention and compassion mediation training on amygdala response to emotional stimuli in an ordinary, non-meditative state. Frontiers in Human Neuroscience.

[CR7] Desikan RS, Segonne F, Fischl B, Quinn BT, Dickerson BC, Blacker D (2006). An automated labeling system for subdividing the human cerebral cortex on MRI scans into gyral based regions of interest. Neuroimage.

[CR8] Farb NA, Segal ZV, Mayberg H, Bean J, McKeon D, Fatima Z (2007). Attending to the present: mindfulness meditation reveals distinct neural modes of self-reference. Social Cognitive and Affective Neuroscience.

[CR9] Fischl B, Salat DH, Busa E, Albert M, Dieterich M, Haselgrove C (2002). Whole brain segmentation: automated labeling of neuroanatomical structures in the human brain. Neuron.

[CR10] Fox KC, Nijeboer S, Dixon ML, Floman JL, Ellamil M, Rumak SP (2014). Is meditation associated with altered brain structure? A systematic review and meta-analysis of morphometric neuroimaging in meditation practitioners. Neuroscience and Biobehavioral Reviews.

[CR11] FreeSurfer (2013). FreeSurfer Software Suite. http://surfer.nmr.mgh.harvard.edu. Accessed 01–05-2015.

[CR12] Friedewald WT, Levy RI, Fredrickson DS (1972). Estimation of the concentration of low-density lipoprotein cholesterol in plasma, without use of the preparative ultracentrifuge. Clinical Chemistry.

[CR13] Goldin PR, Gross JJ (2010). Effects of mindfulness-based stress reduction (MBSR) on emotion regulation in social anxiety disorder. Emotion.

[CR14] Gotink, R. A., Chu, P., Busschbach, J. J., Benson, H., Fricchione, G. L., & Hunink, M. M. (2015). Standardised mindfulness-based interventions in healthcare: an overview of systematic reviews and meta-analyses of RCTs. *PLoS One, 10*(4). 10.1371/journal.pone.0124344 PMC4400080 25881019

[CR15] Grant JA, Courtemanche J, Duerden EG, Duncan GH, Rainville P (2010). Cortical thickness and pain sensitivity in zen meditators. Emotion.

[CR16] Grobbee DE, Hoes AW (2015). Clinical epidemiology: principles, methods and applications for clinical research.

[CR17] Gu J, Strauss C, Bond R, Cavanagh K (2015). How do mindfulness-based cognitive therapy and mindfulness-based stress reduction improve mental health and wellbeing? A systematic review and meta-analysis of mediation studies. Clinical Psychology Review.

[CR18] Hofman A, Brusselle GGO, Murad SD, van Duijn CM, Franco OH, Goedegebure A (2015). The Rotterdam study: 2016 objectives and design update. European Journal of Epidemiology.

[CR19] Holzel BK, Ott U, Gard T, Hempel H, Weygandt M, Morgen K (2008). Investigation of mindfulness meditation practitioners with voxel-based morphometry. Social Cognitive and Affective Neuroscience.

[CR20] Holzel BK, Carmody J, Evans KC, Hoge EA, Dusek JA, Morgan L (2010). Stress reduction correlates with structural changes in the amygdala. Social Cognitive and Affective Neuroscience.

[CR21] Holzel BK, Carmody J, Vangel M, Congleton C, Yerramsetti SM, Gard T (2011). Mindfulness practice leads to increases in regional brain gray matter density. Psychiatry Research.

[CR22] Holzel BK, Hoge EA, Greve DN, Gard T, Creswell JD, Brown KW (2013). Neural mechanisms of symptom improvements in generalized anxiety disorder following mindfulness training. NeuroImage Clinical.

[CR23] Ikram MA, van der Lugt A, Niessen WJ, Krestin GP, Koudstaal PJ, Hofman A (2011). The Rotterdam scan study: design and update up to 2012. European Journal of Epidemiology.

[CR24] Jones DK, Simmons A, Williams SCR, Horsfield MA (1999). Non-invasive assessment of axonal fiber connectivity in the human brain via diffusion tensor MRI. Magnetic Resonance in Medicine.

[CR25] Kang DH, Jo HJ, Jung WH, Kim SH, Jung YH, Choi CH (2013). The effect of meditation on brain structure: cortical thickness mapping and diffusion tensor imaging. Social Cognitive and Affective Neuroscience.

[CR26] Kolb B, Wishaw IQ (2009). Fundamentals of human neuropsychology.

[CR27] Lange C, Irle E (2004). Enlarged amygdala volume and reduced hippocampal volume in young women with major depression. Psychological Medicine.

[CR28] Lanteaume L, Khalfa S, Regis J, Marquis P, Chauvel P, Bartolomei F (2007). Emotion induction after direct intracerebral stimulations of human amygdala. Cerebral Cortex.

[CR29] Lazar SW, Kerr CE, Wasserman RH, Gray JR, Greve DN, Treadway MT (2005). Meditation experience is associated with increased cortical thickness. Neuroreport.

[CR30] Lewinsohn PM, Seeley JR, Roberts RE, Allen NB (1997). Center for Epidemiologic Studies Depression Scale (CES-D) as a screening instrument for depression among community-residing older adults. Psychology and Aging.

[CR31] Lin, K.-Y., Hu, Y.-T., Chang, K.-J., Lin, H.-F., & Tsauo, J.-Y. (2011). Effects of yoga on psychological health, quality of life, and physical health of patients with cancer: a meta-analysis. *Evidence-Based Complementary and Alternative Medicine, 2011*. 10.1155/2011/659876 PMC3062158 21437197

[CR32] Luders E, Toga AW, Lepore N, Gaser C (2009). The underlying anatomical correlates of long-term meditation: larger hippocampal and frontal volumes of gray matter. Neuroimage.

[CR33] Lutz A, McFarlin DR, Perlman DM, Salomons TV, Davidson RJ (2013). Altered anterior insula activation during anticipation and experience of painful stimuli in expert meditators. Neuroimage.

[CR34] Markowitsch HJ (1998). Differential contribution of right and left amygdala to affective information processing. Behavioural Neurology.

[CR35] Modinos G, Ormel J, Aleman A (2010). Individual differences in dispositional mindfulness and brain activity involved in reappraisal of emotion. Social Cognitive and Affective Neuroscience.

[CR36] Mosterd A, Cost B, Hoes AW, de Bruijne MC, Deckers JW, Hofman A (2001). The prognosis of heart failure in the general population: the Rotterdam study. European Heart Journal.

[CR37] Murakami H, Nakao T, Matsunaga M, Kasuya Y, Shinoda J, Yamada J (2012). The structure of mindful brain. PLoS One.

[CR38] Murray, E. A. (2009). *The human amygdala, amygdala function in positive reinforcement*. Guilford Press.

[CR39] Pagnoni G, Cekic M (2007). Age effects on gray matter volume and attentional performance in Zen meditation. Neurobiology of Aging.

[CR40] Pearl J (2009). Causal inference in statistics: an overview. Statistics Surveys.

[CR41] Phelps EA, Delgado MR, Nearing KI, LeDoux JE (2004). Extinction learning in humans: role of the amygdala and vmPFC. Neuron.

[CR42] Pickut BA, Van Hecke W, Kerckhofs E, Marien P, Vanneste S, Cras P (2013). Mindfulness based intervention in Parkinson’s disease leads to structural brain changes on MRI: a randomized controlled longitudinal trial. Clinical Neurology & Neurosurgery.

[CR43] Reuter M, Schmansky NJ, Rosas HD, Fischl B (2012). Within-subject template estimation for unbiased longitudinal image analysis. Neuroimage.

[CR44] Spinhoven PH, Ormel J, Sloekers PPA, Kempen GIJM, Speckens AEM, Hemert AMV (1997). A validation study of the Hospital Anxiety and Depression Scale (HADS) in different groups of Dutch subjects. Psychological Medicine.

[CR45] Statistics, U. I. f. (2012). International standard classification of education ISCED 2011.

[CR46] Taren, A. A., Creswell, J., & Gianaros, P. J. (2013). Dispositional mindfulness co-varies with smaller amygdala and caudate volumes in community adults. *PLoS One, 8*(5). 10.1371/journal.pone.0064574 PMC3661490 23717632

[CR47] Tomasino, B., Fregona, S., Skrap, M., & Fabbro, F. (2013). Meditation related activations are modulated by the practices needed to obtain it and by the expertise: an ALE meta-analysis study. [Original Research]. *Frontiers in Human Neuroscience, 6*, 10.3389/fnhum.2012.00346. 10.3389/fnhum.2012.00346 PMC3539725 23316154

[CR48] Tottenham, N., & Sheridan, M. A. (2009). A review of adversity, the amygdala and the hippocampus: a consideration of developmental timing. *Frontiers in Human Neuroscience, 3*. 10.3389/neuro.09.068.2009 PMC2813726 20161700

[CR49] Vestergaard-Poulsen P, van Beek M, Skewes J, Bjarkam CR, Stubberup M, Bertelsen J (2009). Long-term meditation is associated with increased gray matter density in the brain stem. Neuroreport.

[CR50] Wolsko PM, Eisenberg DM, Davis RB, Phillips RS (2004). Use of mind-body medical therapies. J Gen Intern Med.

